# Genicular nerve block in people with total knee arthroplasty: A systematic review and meta-analysis

**DOI:** 10.1097/MD.0000000000044362

**Published:** 2025-09-12

**Authors:** Yu-Shan Chang, Shao-An Lee, Han-Lin Wang, Wei-Cheng Liao

**Affiliations:** a Department of Physical Medicine and Rehabilitation, Changhua Christian Hospital, Changhua, Taiwan; b Department of Physical Medicine and Rehabilitation, Lo-Hsu Medical Foundation, Inc., Lotung Poh-Ai Hos-pital, Yilan, Taiwan; c Department of Pediatrics, Changhua Christian Hospital, Changhua, Taiwan; d Department of Physical Medicine and Rehabilitation, Nantou Christian Hospital, Nantou, Taiwan.

**Keywords:** knee function, pain control, pain management, postoperative pain

## Abstract

**Background:**

No systematic review has assessed genicular nerve blocks (GNB) efficacy in total knee arthroplasty (TKA) widely. This study evaluated its effectiveness in patients undergoing TKA.

**Methods:**

We conducted a Preferred Reporting Items for Systematic Reviews and Meta-Analyses (PRISMA)-guided systematic review and meta-analysis (PROSPERO CRD42024593742) of randomized controlled trials on GNB in TKA. Searches of PubMed, Embase, and Cochrane Library (July 2014–July 2024) included English-only studies. Risk of bias was assessed using Cochrane 2.0, and random-effects models were applied. No funding was received.

**Results:**

This meta-analysis included 6 randomized controlled trials (335 patients; 169 GNB). Primary outcomes were pain scores (Visual Analog Scale), morphine use, and knee flexion. GNB significantly lowered pain at all time points: 4 hours (rest mean difference [MD]: –3.38, 95% confidence interval [CI]: –6.62 to –0.15, *P* = .04; activity MD: –3.91, 95% CI: –4.16 to –3.66, *P* < .00001), 8 hours (rest MD: –2.88, *P* = .01; activity MD: –3.58, *P* = .01), 12 hours (rest MD: –2.27, *P* = .004; activity MD: –2.33, *P* = .02), and 24 hours (rest MD: –1.40, *P* < .00001; activity MD: –2.42, *P* < .00001). However, GNB did not significantly affect knee flexion (MD: 19.86, 95% CI: –7.26 to 46.98, *P* = .15) or morphine use (SMD: 0.36, 95% CI: –2.22 to 2.93, *P* = .79). Subgroup analysis confirmed pain reduction with GNB alone or combined with other blocks at 12 and 24 hours: rest (MD: –1.10, *P* = .0001; MD: –2.85, *P* < .0001) and activity (MD: –1.30, *P* < .0001; MD: –4.00, *P* < .00001).

**Conclusion:**

GNB reduces early postoperative pain after TKA without improving function or opioid use. Limitations include few trials, English-only data, short follow-up, and no complication assessment. Further research should confirm long-term efficacy and safety.

## 1. Introduction

Total knee arthroplasty (TKA) remains one of the most frequently performed orthopedic procedures, with approximately 1,065,000 individuals undergoing this surgery in the United States in 2020. This figure is anticipated to rise to 3,416,000 by 2040.^[1]^ Despite its widespread use, roughly 19% of patients undergoing primary TKA report dissatisfaction with the outcomes.^[2]^ Factors contributing to dissatisfaction include unmet preoperative expectations, in-sufficient functional improvement, and inadequate pain relief postoperatively.^[3]^

Peripheral nerve blocks were regarded as the preferred alternative to epidural blocks.^[4]^ Genicular nerve blocks (GNB), which utilize a variety of agents, had emerged as a novel treatment option, with numerous case reports and series demonstrating temporary relief for patients.^[5,6]^ Motor-sparing peripheral nerve blocks facilitated early mobility, provided effective pain control, and minimized the risk of opioid-related adverse effects, making them particularly suitable for outpatient and ambulatory lower-extremity procedures.^[7]^ However, the efficacy of GNB in the TKA remains incompletely defined. Several studies have reported inconsistent results regarding pain relief and functional recovery. Some studies suggest GNB provides significant analgesia, while others report minimal or no benefit.^[6–11]^

To our knowledge, no formal systematic review has examined the efficacy of GNB within the context of TKA. Accordingly, we undertook a systematic review of randomized controlled trials (RCTs) to assess the effectiveness of GNB in patients undergoing TKA comprehensively. We hypothesized that GNB significantly reduces postoperative pain scores and improves knee function in TKA patients.

## 2. Materials and methods

### 2.1. Search strategy

A thorough search was conducted following the 2020 guidelines of the Preferred Reporting Items for Systematic Reviews and Meta-Analyses.^[12]^ This systematic review was conducted following a protocol registered prospectively with PROSPERO (registration number: CRD42024593742). We performed a comprehensive search to identify full-text RCTs assessing the efficacy of GNBs in relation to TKA. The literature search encompassed multiple major electronic databases, including: PubMed, Embase, and the Cochrane Library. All of the studies published between July 31, 2014, and July 31, 2024, were considered. The search terms used included: (“total knee arthroplasty” OR “total knee replacement” OR “knee arthroplasty, total” OR “arthroplasty, total knee”) AND (“genicular nerve block” OR “genicular nerve blockade” OR “chemical genicular neurolysis” OR “chemical genicular neurolysis” OR “genicular neurolysis, chemical” OR “genicular chemodenervation” OR “genicular chemodenervations” “genicular nerve analgesia” OR “genicular nerve radiofrequency ablation”). The search was limited to articles in the English language only. In addition, we reviewed reference lists of relevant articles to identify additional studies that might have been missed during the initial search. All articles, including both primary studies and review studies, as well as their references, were independently assessed in duplicate. The detailed search strategy is available in Table S1, Supplemental Digital Content, https://links.lww.com/MD/P899.

### 2.2. Inclusion criteria

Studies were included in this review if they satisfied the following criteria: they were full-text RCTs; participants were aged 18 years or older; participants underwent TKA with the application of GNB; and participants may have received other analgesic agents in addition to the GNB.

### 2.3. Exclusion criteria

The exclusion criteria were as follows: non-English publications; and studies involving participants younger than 18 years.

### 2.4. Primary outcome

Data extracted included pain levels at rest and during movement, morphine consumption, and knee function. Pain levels were assessed subjectively utilizing the Visual Analog Scale (VAS), which ranges from 0 to 10, for both resting and movement conditions. Objective pain assessment included quantifying morphine consumption over 48 hours (measured in milligrams (mg) or milliequivalents (mEq)). Knee function was evaluated by measuring the degree of knee flexion.

### 2.5. Data extraction

We collected the following information independently: study design, details of the intervention, values for designated outcomes, and the follow-up intervals or sessions. In multiple-arm studies, we combined similar eligible groups within the intervention or control arms for pairwise comparisons.

### 2.6. Bias assessment and quality classification

The risk of bias presented in the included studies was systematically assessed using the Cochrane Risk of Bias tool 2.0, covering 6 domains: randomization process, deviations from intended interventions, missing outcome data, outcome measurement, selection of reported results, and overall bias.^[13]^ For each domain, the risk was categorized as “low,” “unclear,” or “high.”

### 2.7. Statistical analysis

We used Review Manager (RevMan 5.3, The Nordic Cochrane Centre, The Cochrane Collaboration, Copenhagen, Denmark) software to calculate the pooled mean difference (MD) and standard deviation (SD) for continuous outcomes. The effect size in the combined analysis was represented by a 95% confidence interval (CI). The SD was estimated using the range divided by 4, and median values were converted to means as previously described.^[9]^ Heterogeneity was assessed using χ² and *I*² tests. Due to heterogeneity among studies (including differences in design, patient populations, and other variables) a random-effects model was employed. Subgroup analyses compared patients receiving GNB alone versus those receiving GNB with additional block methods. Statistical significance was considered achieved when the *P*-value was <.05.

### 2.8. Ethical reviews

We do not need ethical review due to this is a systematic review based on previous studies.

## 3. Results

### 3.1. Study identification and selection

**Figure 1.**
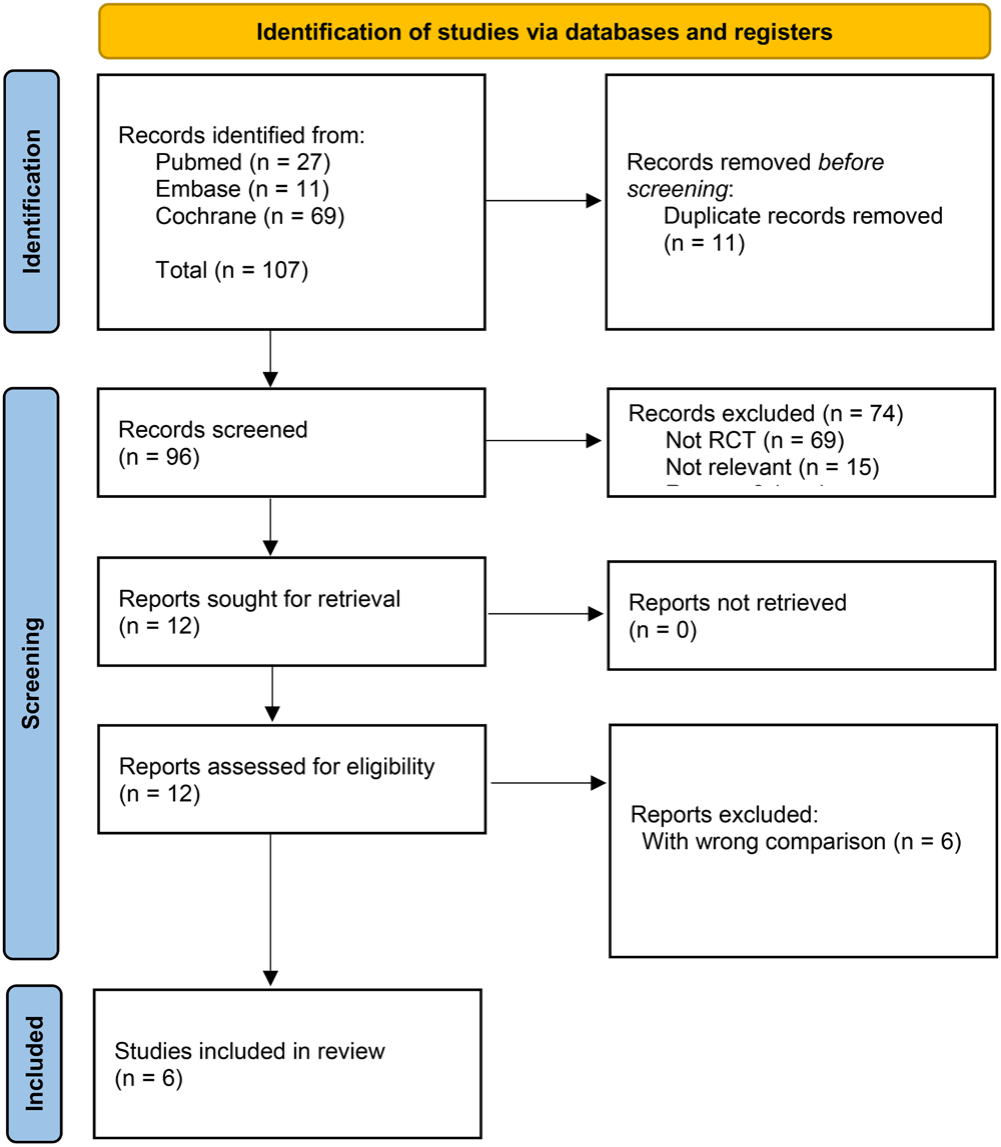
Flow diagram describing the screening and review processes of the meta-analysis.

A comprehensive literature search identified 107 relevant studies. After excluding 11 duplicates, 96 studies remained for screening. Title and abstract review led to the elimination of 74 studies due to reasons such as non-randomized controlled trial design or lack of relevance, leaving 12 studies for full-text evaluation. Upon thorough assessment of these full texts, 6 additional studies were excluded because of inappropriate comparisons (The precise reasons are showed in Table S2, Supplemental Digital Content, https://links.lww.com/MD/P899).^[6–11]^ Ultimately, 6 RCTs were included in the final meta-analysis (Fig. 1).^[14–19]^

Six RCTs were included in this meta-analysis, involving a total of 335 patients divided into 2 groups: 169 patients received a GNB, while 166 served as controls. Detailed patient characteristics from all included studies are presented in Table 1. The trials were published between July 31, 2014, and July 31, 2024, with sample sizes ranging from 40 to 88 participants. One study administered the GNB 2 to 6 weeks before TKA, while another did so 2 to 4 weeks before the procedure. The remaining studies performed the block immediately before surgery. Two studies utilized radiofrequency ablation for nerve blockage, whereas the others employed local anesthetics. The efficacy of the GNB was evaluated in 3 studies using the Numeric Rating Scale and in another 3 using the VAS. Furthermore, only 3 studies measured morphine consumption, and 3 assessed knee function through the degree of knee flexion.

**Table 1:** Characteristics of the included studies.

References	Participants (E/C)	GNB methods	Timing of GNB
Walega^[14]^	35/32	Radiofrequency ablation	2–6 wk prior to TKA
Rambhla^[15]^	20/20	Local anesthetics	Just before TKA
Mishra^[16]^	30/30	Radiofrequency ablation	2–6 wk prior to TKA
Akesen^[17]^	20/20	Local anesthetics	Just before TKA
Kampitak^[18]^	44/44	Local anesthetics	Just before TKA
Dundar^[19]^	20/20	Local anesthetics	Just before TKA

C = control, E = experimental, GNB = genicular nerve block, TKA = total knee arthroplasty.

### 3.2. Study identification and selection

**Figure 2.**
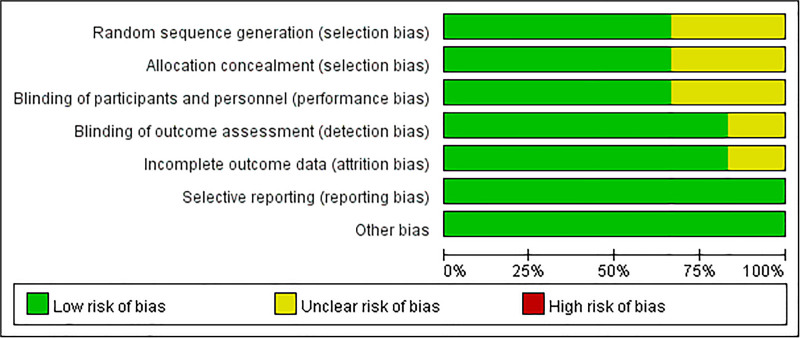
Summary of quality assessment of studies included in the meta-analysis using Cochrane risk of bias 2 tool.

**Figure 3.**
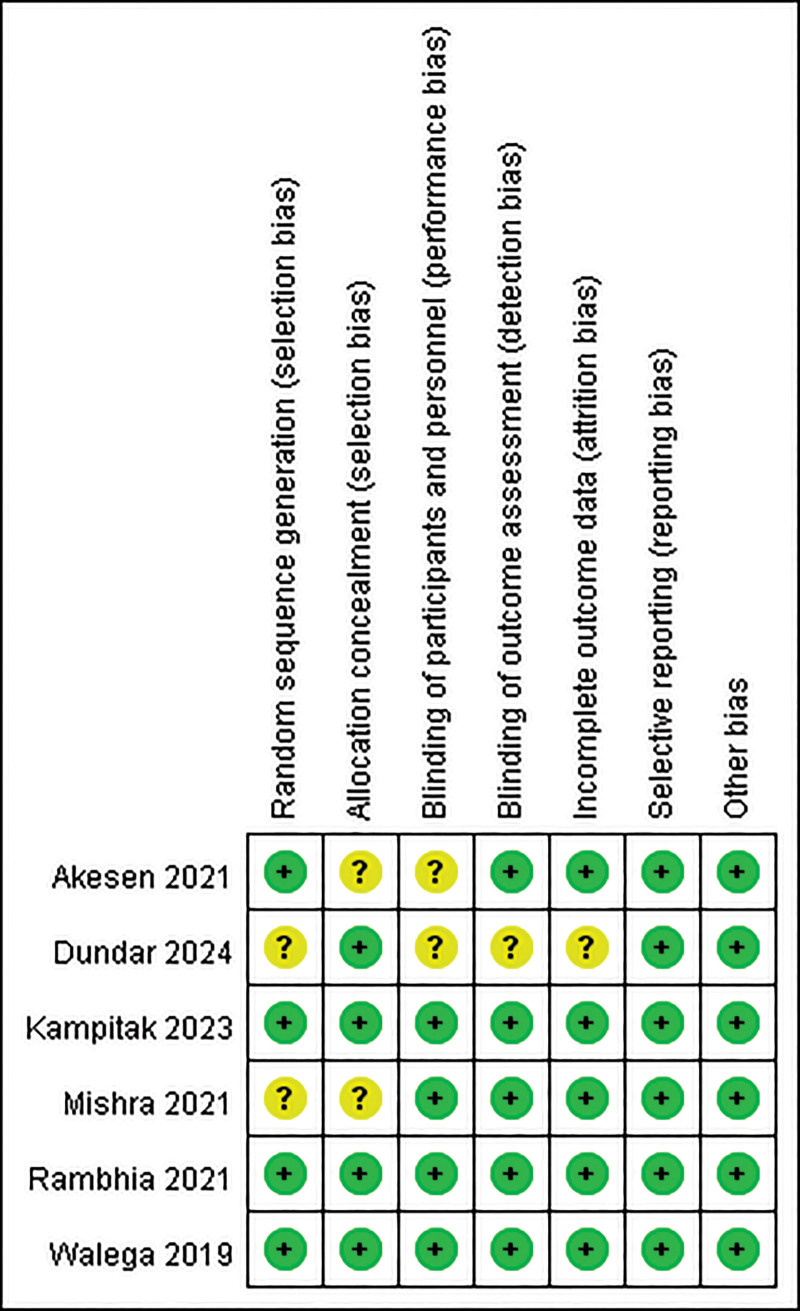
Summary of quality assessment of studies included in the meta-analysis using Cochrane risk of bias 2 tool.

The risk of bias associated with this study is illustrated in Figures 2 and 3.

#### 3.2.1. Random sequence generation

Only 2 trials exhibited an unclear risk of bias concerning random sequence generation.^[16,19]^ Both studies demonstrated significant differences in baseline characteristics between the experimental and control groups. In 1 study, the genicular nerve group reported significantly lower scores on the Knee Society Score, Oxford Knee Score, and Western Ontario and McMaster Universities Osteoarthritis Index in comparison to the control group (*P* < .001).^[19]^ The other experimental group was characterized by a younger demographic, a higher body mass index, and a lower proportion of female participants.^[16]^

#### 3.2.2. Allocation concealment

Two trials exhibited an unclear risk of bias regarding allocation concealment, as they failed to provide any description of this aspect.^[16,17]^

#### 3.2.3. Blinding of participants and personnel

Two studies failed to disclose information regarding the blinding of participants, leading to an ambiguous assessment of the risk of bias associated with the blinding of both participants and personnel.^[17,19]^

#### 3.2.4. Blinding of outcome assessment

Two studies exhibited an unclear risk of bias in this section.^[17,19]^ One study failed to describe the blinding process for outcome assessment.^[17]^ In contrast, the other study indicated that the surgical team was not blinded to the pain management methodology; however, the evaluation of intraoperative and postoperative data was conducted by the anesthesiology team, which was separate from the surgical team.^[19]^

#### 3.2.5. Incomplete outcome data

Only 1 trial demonstrated an unclear risk of bias related to incomplete outcome data, as it reported 6 participants lost to follow-up in both the experimental and control groups. However, the trial did not offer any explanations for these losses.^[19]^

#### 3.2.6. Selective reporting

All trials demonstrated a minimal risk of bias concerning selective reporting, as all pertinent outcomes were reported appropriately.

#### 3.2.7. Other bias

None of the trials indicated the presence of additional sources of bias.

### 3.3. Outcome

#### 3.3.1. Pain scores at 4 and 8 hours

**Figure 4.**
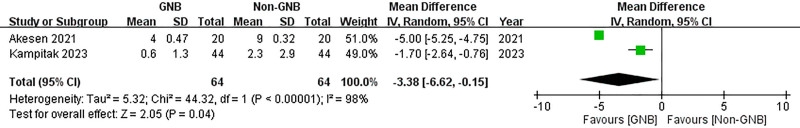
Forest plot of the effects of GNB at 4 hours post-TKA on pain score (VAS) at rest. GNB = genicular nerve blocks, TKA = total knee arthroplasty, VAS = Visual Analog Scale.

**Figure 5.**
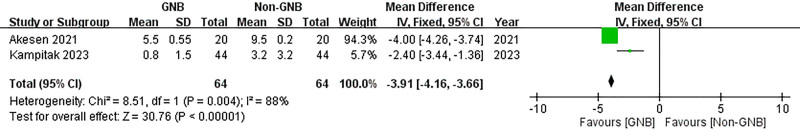
Forest plot of the effects of GNB at 4 hours post-TKA on pain score (VAS) during activity. GNB = genicular nerve blocks, TKA = total knee arthroplasty, VAS = Visual Analog Scale.

**Figure 6.**
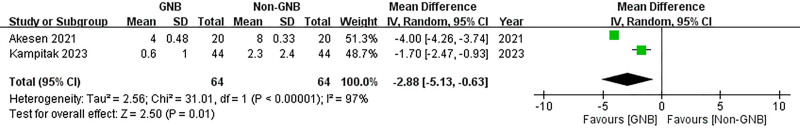
Forest plot of the effects of GNB at 8 hours post-TKA on pain score (VAS) at rest. GNB = genicular nerve blocks, TKA = total knee arthroplasty, VAS = Visual Analog Scale.

**Figure 7.**
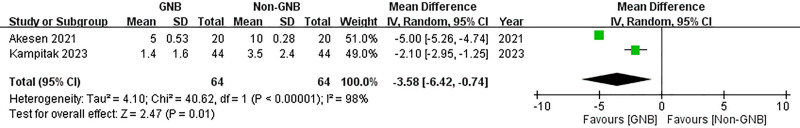
Forest plot of the effects of GNB at 8 hours post-TKA on pain score (VAS) during activity. GNB = genicular nerve blocks, TKA = total knee arthroplasty, VAS = Visual Analog Scale.

Two studies (n = 128 participants) assessed pain using the VAS at rest and during activity at 4 and 8 hours post-TKA. Patients who received GNB had significantly lower VAS pain scores at both time points and under both conditions compared with controls [at 4 hours (rest): MD = –3.38, 95% CI: –6.62 to –0.15; *P* = .04; at 4 hours (activity): MD = –3.91, 95% CI: –4.16 to –3.66; *P* < .00001; at 8 hours (rest): MD = –2.88, 95% CI: –5.13 to –0.63; *P* = .01; At 8 hours (activity): MD = –3.58, 95% CI: –6.42 to –0.74; *P* = .01] (Figs. 4–7).

#### 3.3.2. Pain scores at 12 and 24 hours

**Figure 8.**
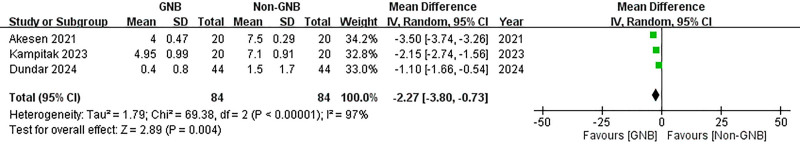
Forest plot of the effects of GNB at 12 hours post-TKA on pain score (VAS) at rest. GNB = genicular nerve blocks, TKA = total knee arthroplasty, VAS = Visual Analog Scale.

**Figure 9.**
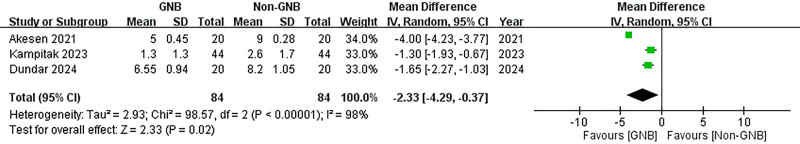
Forest plot of the effects of GNB at 12 hours post-TKA on pain score (VAS) during activity. GNB = genicular nerve blocks, TKA = total knee arthroplasty, VAS = Visual Analog Scale.

**Figure 10.**
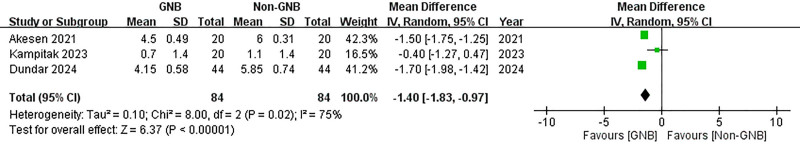
Forest plot of the effects of GNB at 12 hours post-TKA on pain score (VAS) during activity. GNB = genicular nerve blocks, TKA = total knee arthroplasty, VAS = Visual Analog Scale.

**Figure 11.**
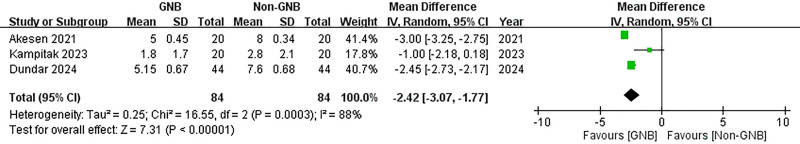
Forest plot of the effects of GNB at 24 hours post-TKA on pain score (VAS) during activity. GNB = genicular nerve blocks, TKA = total knee arthroplasty, VAS = Visual Analog Scale.

Three studies (n = 168 participants) evaluated VAS pain scores at rest and during activity at 12 and 24 hours post-TKA. The GNB group demonstrated significantly lower pain scores than controls at both time points and under both resting and active conditions [at 12 hours (rest): MD = –2.27, 95% CI: –3.80 to –0.73; *P* = .004; at 12 hours (activity): MD = –2.33, 95% CI: –4.29 to –0.37; *P* = .02; at 24 hours (rest): MD = –1.40, 95% CI: –1.83 to –0.97; *P* < .00001; at 24 hours (activity): MD = –2.42, 95% CI: –3.07 to –1.77; <.00001] (Figs. 8–11).

#### 3.3.3. Knee flexion

**Figure 12.**
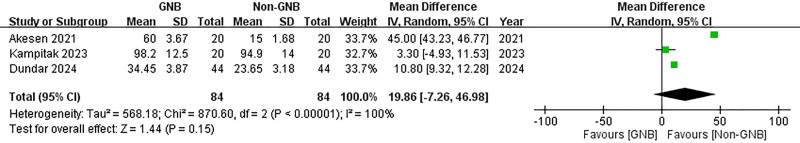
Forest plot of the effects of GNB on knee flexion (degree). GNB = genicular nerve blocks.

The degree of knee flexion was also assessed, with no significant differences observed between the GNB and control groups (MD = 19.86, 95% CI: –7.26 to 46.98; *P* = .15) (Fig. 12).

#### 3.3.4. Morphine consumption

**Figure 13.**
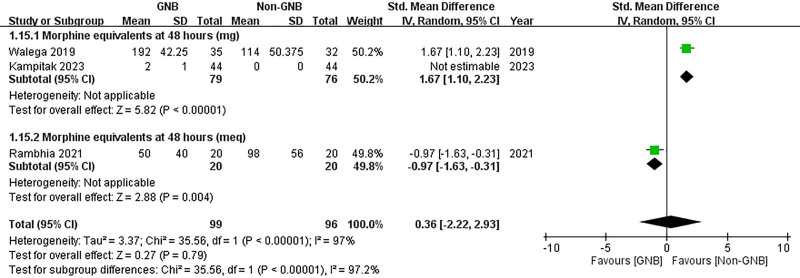
Forest plot of the effects of GNB on morphine consumption. GNB = genicular nerve blocks.

Three studies (n = 195 participants) measured morphine consumption at 48 hours post-TKA. Two studies quantified consumption in mg, while 1 used mEq. The study using mEq reported decreased morphine consumption in the GNB group (SMD = –0.97, 95% CI: –1.63 to −0.31; *P* = .004), whereas the studies measuring mg indicated lower morphine consumption in the control group at 48 hours (SMD = 1.67, 95% CI: 1.10 to 2.23; *P* < .00001). Pooled analysis showed no significant difference in morphine consumption between the GNB and control groups (SMD = 0.36, 95% CI: −2.22 to 2.93; *P* = .79) (Fig. 13).

#### 3.3.5. Comparison of pain reduction at rest versus movement

**Figure 14.**
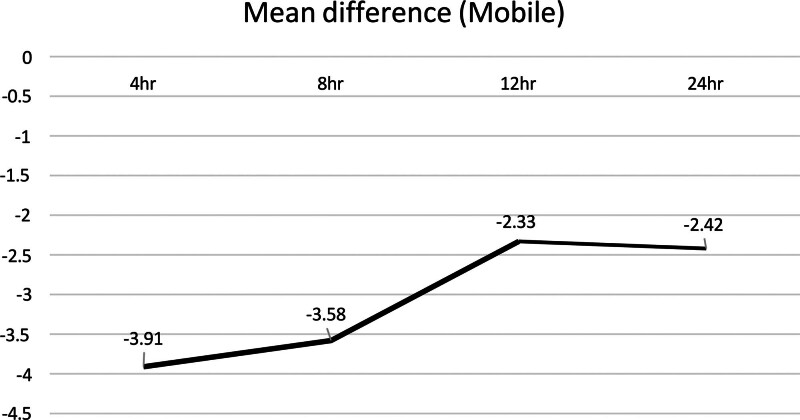
Line graft of the mean difference in pain during movement between the experimental and control groups at various time points post-TKA. TKA = total knee arthroplasty.

**Figure 15.**
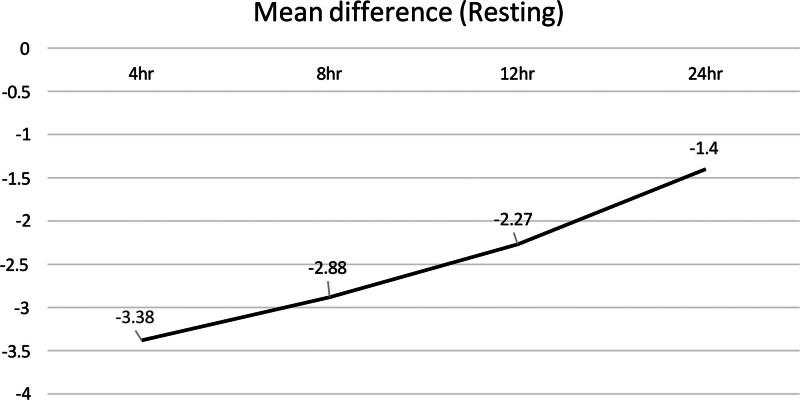
Line graft of the mean difference in pain at rest between the experimental and control groups at various time points post-TKA. TKA = total knee arthroplasty.

GNB was more effective in reducing pain during movement than at rest following TKA [mobile: MD = –3.91 (at 4 hours), −3.58 (at 8 hours), −2.33 (at 12 hours), −2.42 (at 24 hours); resting: MD = –3.38 (at 4 hours), −2.88 (at 8 hours), −2.27 (at 12 hours), −1.4 (at 24 hours)] (Figs. 14 and 15).

#### 3.3.6. Subgroup analysis

**Figure 16.**
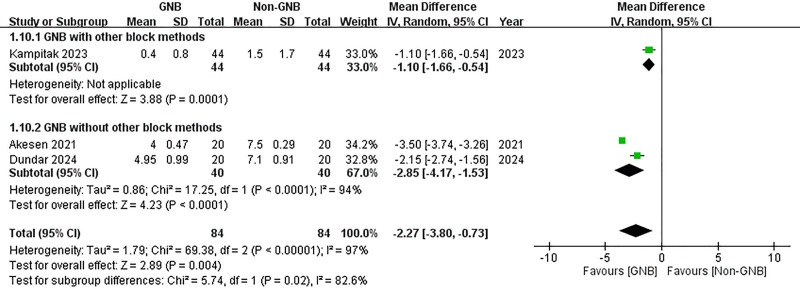
Forest plot of the subgroup analysis. Compare participants using GNB with those also using other block methods. The effects of GNB at 12 hours post-TKA on pain score (VAS) at rest. GNB = genicular nerve blocks, TKA = total knee arthroplasty, VAS = Visual Analog Scale.

**Figure 17.**
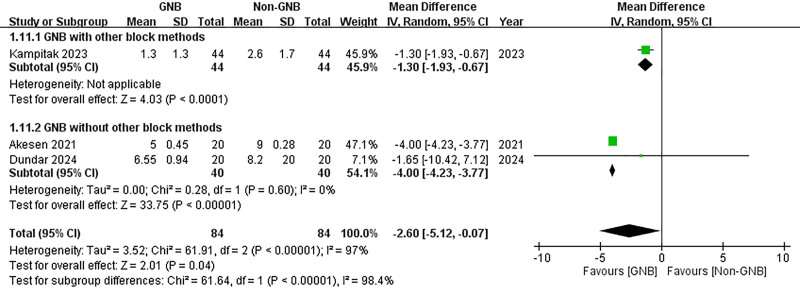
Forest plot of the subgroup analysis. Compare participants using GNB with those also using other block methods. The effects of GNB at 12 hours post-TKA on pain score (VAS) during activity. GNB = genicular nerve blocks, TKA = total knee arthroplasty, VAS = Visual Analog Scale.

**Figure 18.**
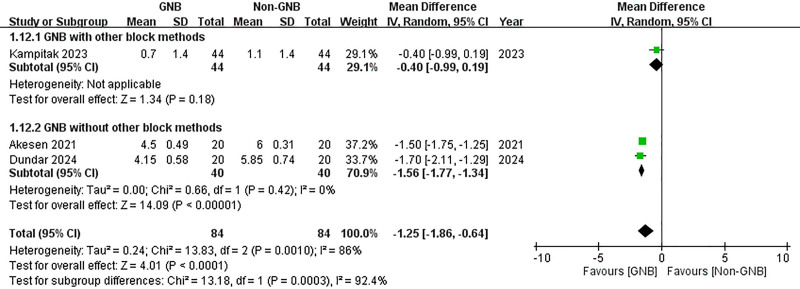
Forest plot of the subgroup analysis. Compare participants using GNB with those also using other block methods. The effects of GNB at 24 hours post-TKA on pain score (VAS) at rest. GNB = genicular nerve blocks, TKA = total knee arthroplasty, VAS = Visual Analog Scale.

**Figure 19.**
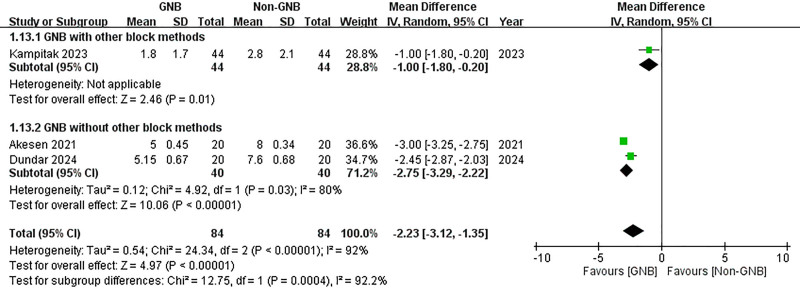
Forest plot of the subgroup analysis. Compare participants using GNB with those also using other block methods. The effects of GNB at 24 hours post-TKA on pain score (VAS) during activity. GNB = genicular nerve blocks, TKA = total knee arthroplasty, VAS = Visual Analog Scale.

**Figure 20.**
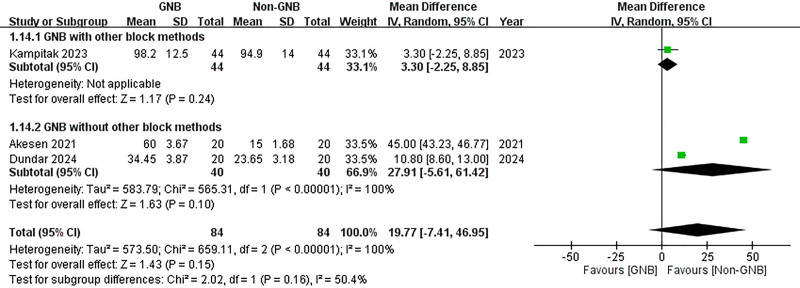
Forest plot of the subgroup analysis. Compare participants using GNB with those also using other block methods. The effects of GNB on knee flexion (degree). GNB = genicular nerve blocks.

A subgroup analysis was conducted to compare participants who received only the GNB with those who also underwent additional block methods. In both subgroups, significant reductions in VAS scores were observed at 12 hours post-TKA for both pain at rest and during activity (with additional block methods (rest): MD = –1.10, 95% CI: –1.66 to –0.54; *P* = .0001; without additional block methods (rest): MD = –2.85, 95% CI: –4.17 to –1.53; *P* < .0001; with additional block methods (activity): MD = –1.30, 95% CI: –1.96 to –0.67; *P* < .0001; without additional block methods (activity): MD = –4.00, 95% CI: –4.23 to –3.77; *P* < .00001) (Figs. 16 and 17). However, at 24 hours post-TKA, participants who received additional block methods exhibited no significant difference in resting VAS scores when comparing those who received the GNB with those who did not (MD = –0.40, 95% CI: –0.99 to 0.19; *P* = .18) (Fig. 18). Conversely, a significant difference was observed in mobile VAS scores at this time point (MD = –1.00, 95% CI: –1.80 to −0.20; *P* = .01) (Fig. 19). Furthermore, there were no significant differences in the degree of knee flexion between participants in either subgroup (with additional block methods: MD = 3.30, 95% CI: –2.25 to 8.85; *P* = .24; without additional methods: MD = 27.91, 95% CI: –5.61 to 61.42; *P* = .10) (Fig. 20).

## 4. Discussion

This systematic literature review examined outcome reporting in RCTs related to GNB in TKA. A total of 6 studies, encompassing 128 participants, were included in the analysis. The results suggest that GNB are effective in reducing pain, as assessed by the VAS, for a duration of at least 24 hours during both rest and movement. Nevertheless, the intervention did not show a statistically significant effect on knee flexion or morphine consumption.

### 4.1. Meta-analytic value and resolution of conflicting evidence

Individual studies examining GNB in TKA have reported inconsistent results, with some demonstrating significant analgesic benefits while others showed minimal effects.^[6–11,14–19]^ Our comprehensive analysis resolves these apparent contradictions by demonstrating that the pooled effect consistently favors GNB across all time intervals and circumstances. The heterogeneity observed in individual studies actually provides valuable insights when synthesized.^[14–19]^ This heterogeneity largely stems from methodological variations (including timing of block administration, technique used, and concurrent interventions) rather than fundamental differences in GNB efficacy. By systematically analyzing these sources of variation through subgroup analysis, our meta-analysis transforms apparent inconsistency into clinically relevant guidance about optimal GNB implementation.

This meta-analysis fills important gaps in the current evidence base that single studies have been unable to address. First, it establishes GNB as an effective component of multimodal analgesia with consistent effect sizes across diverse patient populations. Additionally, it clarifies that while GNB provides excellent analgesia, expectations for functional improvement and opioid sparing should be tempered. Finally, it provides the first systematic evidence concerning the best timing and combination approaches for the application of GNB.

### 4.2. Meta-analytic value and resolution of conflicting evidence

Our pooled analysis demonstrates that GNB provides significant pain relief in the early postoperative period. Notably, the sustained reduction in pain intensity at 12 and 24 hours indicates its prolonged analgesic effect. This may facilitate earlier patient mobilization and engagement in rehabilitation. Importantly, a distinct contribution of our meta-analysis is the consistent finding that GNB is more effective in alleviating movement-related pain than pain at rest at all evaluated time points. This distinction carries significant clinical implications, as movement-related pain is a critical barrier to early postoperative mobilization and physical therapy.

The findings of this study align with prior research that has established the efficacy of guided nerve blocks in mitigating knee pain.^[20]^ GNB operates by inhibiting sensory nerve conduction in the vicinity of the knee joint, consequently diminishing the transmission of pain signals.^[21]^ Importantly, our investigation revealed that GNB was more effective in alleviating pain during movement compared to at rest, which may promote early rehabilitation and enhance functional recovery in patients.

### 4.3. Functional outcomes and opioid consumption

However, the administration of GNB did not demonstrate a statistically significant effect on knee flexion or morphine consumption. This lack of impact may be attributed to the fact that the recovery of knee joint function is influenced not only by effective pain management but also by additional factors, including surgical techniques, rehabilitation protocols, and individual patient characteristics.^[22]^ Concerning morphine consumption, despite a reduction in pain scores, no significant difference in opioid usage was observed between the groups. This phenomenon may be associated with the implementation of standardized pain management protocols or individual variations in opioid requirements.^[23]^

### 4.4. Clinical importance and our strengths

Clinically, the GNB was recognized as a motor-sparing peripheral nerve block that facilitates early postoperative mobilization, diminishes the risk of complications, and may contribute to a reduction in hospital length of stay.^[24]^ Furthermore, GNB is relatively straightforward to perform, presents minimal complications, and can serve as an important component of multimodal analgesia in TKA.

Compared to previous literature, this is the first meta-analysis to systematically assess GNB exclusively in the context of TKA. Prior studies have either focused on broader categories of peripheral nerve blocks or assessed GNB in chronic pain settings such as osteoarthritis.^[2–5]^ Our meta-analysis consolidates the evidence specifically for the perioperative period and provides pooled effect estimates that increase statistical power and precision. By integrating results across trials, we address inconsistencies in individual findings and offer a more definitive understanding of GNB’s role.

### 4.5. Limitations

This study has several limitations that warrant consideration. First, the systematic review included a limited number of trials. Second, by restricting our analysis to English-language studies, we may have inadvertently introduced a potential publication bias. Third, our examination concentrated exclusively on pain levels within the first 24 hours postoperatively, thereby constraining our understanding of the effects beyond this initial timeframe. Fourth, we calculated the SD (variance) using the formula range/4 and converted median values to means, which may have introduced some bias into our findings. Fifth, we did not explore potential complications associated with the denervation of the genicular nerve. Future studies should assess the safety and potential adverse effects of GNB.

## 5. Conclusion

This systematic review of RCTs indicated that GNB is effective in reducing postoperative pain following TKA, especially during the early postoperative period and movement. However, GNB did not demonstrate a significant impact on knee function or opioid consumption. Despite these limitations, incorporating GNB into multimodal analgesia protocols can improve postoperative pain management, potentially enhancing patient satisfaction and early rehabilitation outcomes. Future research should focus on evaluating the long-term efficacy, safety, and impact of GNB on functional outcomes to further establish its role in pain management after TKA.

## Author contributions

**Conceptualization:** Wei-Cheng Liao.

**Data curation:** Han-Lin Wang.

**Formal analysis:** Yu-Shan Chang, Shao-An Lee, Han-Lin Wang.

**Investigation:** Yu-Shan Chang, Shao-An Lee.

**Methodology:** Yu-Shan Chang, Shao-An Lee.

**Supervision:** Wei-Cheng Liao.

**Writing – original draft:** Yu-Shan Chang, Han-Lin Wang.

**Writing – review & editing:** Yu-Shan Chang, Wei-Cheng Liao.

## Supplementary Material


