# Supplementary material for: Genicular nerve block in people with total knee arthroplasty: A systematic review and meta-analysis
Source: Medicine (Baltimore). 2025 Sep 12;104(37):e44362. doi: 10.1097/MD.0000000000044362 (PMC12440537; doi:10.1097/MD.0000000000044362)
Supplement: Supplementary file 1 [file medi-104-e44362-s001.docx]

Table S.1. Keywords and search results in different databases.

| **Database** | **Keyword** | **Filter** | **Results** |
| --- | --- | --- | --- |
| Pubmed | (“total knee arthroplasty” OR “total knee replacement” OR “knee arthroplasty, total” OR “arthroplasty, total knee”) AND (“genicular nerve block” OR “genicular nerve blockade” OR “chemical genicular neurolysis” OR “chemical genicular neurolysis” OR “genicular neurolysis, chemical” OR “genicular chemodenervation” OR “genicular chemodenervations” “genicular nerve analgesia” OR “genicular nerve radiofrequency ablation”) | NA | 27 |
| Embase |  | NA | 11 |
| Cochrane library |  | Title abstract  Keyword | 69 |

Table. S.2 Excluded studies and reasons

| **Citations** | **Reasons** |
| --- | --- |
| Cuñat, T., et al., Ultrasound-guided genicular nerves block vs. local infiltration analgesia for total knee arthroplasty: a randomised controlled non-inferiority trial. Anaesthesia, 2023. 78(2): p. 188-196. DOI: 10.1111/anae.15909 | Do not compare GNB with non-GNB |
| Qudsi-Sinclair, S., et al., A Comparison of Genicular Nerve Treatment Using Either Radiofrequency or Analgesic Block with Corticosteroid for Pain after a Total Knee Arthroplasty: A Double-Blind, Randomized Clinical Study. Pain Practice, 2017. 17(5): p. 578-588. DOI: 10.1111/papr.12481 | Do not compare GNB with non-GNB |
| Lyman, J.R., et al., Radiofrequency ablation prior to total knee arthroplasty does not improve post-surgical pain or recovery: a double-blinded, multi-center, randomized clinical trial. Ann Jt, 2023. 8: p. 5. DOI: 10.21037/aoj-22-33 | Do not compare GNB with non-GNB |
| Kuttasseri, T.A., et al., Comparison of ultrasound-guided genicular nerve block and knee periarticular infiltration for postoperative pain and functional outcomes in knee arthroplasty in cardiac patients - A randomised trial. Journal of cardiovascular disease research, 2024. 15(2): p. 616‐622. DOI: 10.48047/jcdr.2024.15.02.65 | Do not compare GNB with non-GNB |
| Eid, G.M., S. El Said Shaban, and T.A. Mostafa, Comparison of ultrasound-guided genicular nerve block and knee periarticular infiltration for postoperative pain and functional outcomes in knee arthroplasty - A randomised trial. Indian J Anaesth, 2023. 67(10): p. 885-892. DOI: 10.4103/ija.ija_449_23 | Do not compare GNB with non-GNB |
| Kertkiatkachorn, W., et al., Intraoperative landmark-based genicular nerve block versus periarticular infiltration for postoperative analgesia in total knee arthroplasty: a randomized non-inferiority trial. Regional anesthesia and pain medicine, 2023. DOI: 10.1136/rapm-2023-104563 | Do not compare GNB with non-GNB |
